# Aberrant Cerebral Iron Trafficking Co-morbid With Chronic Inflammation: Molecular Mechanisms and Pharmacologic Intervention

**DOI:** 10.3389/fneur.2022.855751

**Published:** 2022-03-15

**Authors:** Shaina L. Rosenblum, Daniel J. Kosman

**Affiliations:** Department of Biochemistry, Jacobs School of Medicine and Biomedical Sciences, University at Buffalo, Buffalo, NY, United States

**Keywords:** chronic inflammation, iron trafficking, brain iron, neurodegeneration, aging, blood-brain barrier

## Abstract

The redox properties that make iron an essential nutrient also make iron an efficient pro-oxidant. Given this nascent cytotoxicity, iron homeostasis relies on a combination of iron transporters, chaperones, and redox buffers to manage the non-physiologic aqueous chemistry of this first-row transition metal. Although a mechanistic understanding of the link between brain iron accumulation (BIA) and neurodegenerative diseases is lacking, BIA is co-morbid with the majority of cognitive and motor function disorders. The most prevalent neurodegenerative disorders, including Alzheimer's Disease (AD), Parkinson's Disease (PD), Multiple System Atrophy (MSA), and Multiple Sclerosis (MS), often present with increased deposition of iron into the brain. In addition, ataxias that are linked to mutations in mitochondrial-localized proteins (Friedreich's Ataxia, Spinocerebellar Ataxias) result in mitochondrial iron accumulation and degradation of proton-coupled ATP production leading to neuronal degeneration. A comorbidity common in the elderly is a chronic systemic inflammation mediated by primary cytokines released by macrophages, and acute phase proteins (APPs) released subsequently from the liver. Abluminal inflammation in the brain is found downstream as a result of activation of astrocytes and microglia. Reasonably, the iron that accumulates in the brain comes from the cerebral vasculature *via* the microvascular capillary endothelial cells whose tight junctions represent the blood-brain barrier. A premise amenable to experimental interrogation is that inflammatory stress alters both the trans- and para-cellular flux of iron at this barrier resulting in a net accumulation of abluminal iron over time. This review will summarize the evidence that lends support to this premise; indicate the mechanisms that merit delineation; and highlight possible therapeutic interventions based on this model.

## Introduction

A strong correlation between cognitive decline and systemic pathology underlying chronic inflammation has been long recognized (1–4). Chronic inflammation common as one ages can be linked to arthritis, diabetes, cancer and/or chronic bacterial infection including periodontal disease (5). Another metabolic trajectory common to the elderly is brain xsiron accumulation as noted by both post-mortem quantitative analyses (6) and fMRI (7). This brain iron accumulation occurs despite the commonly co-morbid anemia linked also to chronic inflammation (8). Here we review the molecular cell mechanisms that underlie these clinical patterns. This synthesis provides support for the premise that a combination of therapeutic management of the brain iron and oxidative stress exacerbated by chronic inflammation is a mechanistically rational pharmacologic approach in the suppression of cognitive decline.

## Iron Metabolism

Iron is an essential trace element that is necessary for normal cell functioning, but can be harmful in excess. Due to Fenton chemistry, excessive amounts of iron can produce free radicals that can result in oxidative stress and ultimately, cell death (9). Therefore, iron metabolism must be highly regulated within tissues and cells. Iron functions as a co-factor for several enzymatic processes, energy metabolism, myelination, and DNA synthesis (10–12). The brain has critical need for iron due to this organ's high metabolic activity. In this context, maintenance of iron homeostasis is essential so as to avoid iron-dependent oxidative stress leading to neuronal degeneration and death. Healthy, normal brains accumulate iron specifically within the globus pallidus, caudate nucleus, putamen, dentate nucleus, and substantia nigra as shown by postmortem analysis and MRI (13–15). Iron in the brain can be found in several different forms. Ferrous (Fe^2+^) and ferric (Fe^3+^) iron is chelated by small molecules such and citrate and glutathione; Fe^2+^ is bound to the iron chaperones, PCBP1 and 2; and both redox forms are prosthetic groups of a multitude of proteins, both catalytic and structural. As a polyphosphate complex, ferric iron is stored in ferritin. When ferritin is degraded in the lysosome, hemosiderin is produced, which contains both ferrous and ferric iron. Within neurons, neuromelanin forms complexes with metals, including iron, and is found in autolysosomal organelles mostly in neurons of the substantia nigra and locus coeruleus, but in other regions of the human brain as well (16, 17). Cells of the brain do not have direct access to nutrients, including iron, from the systemic circulation because the blood-brain barrier (BBB) separates the brain from the systemic circulation, preventing toxic material transport. Iron transport into the brain occurs at the BBB, which is composed of brain microvascular endothelial cells (BMVECs) and is supported by astrocytes, neurons and microglia (18).

**Figure 1:**
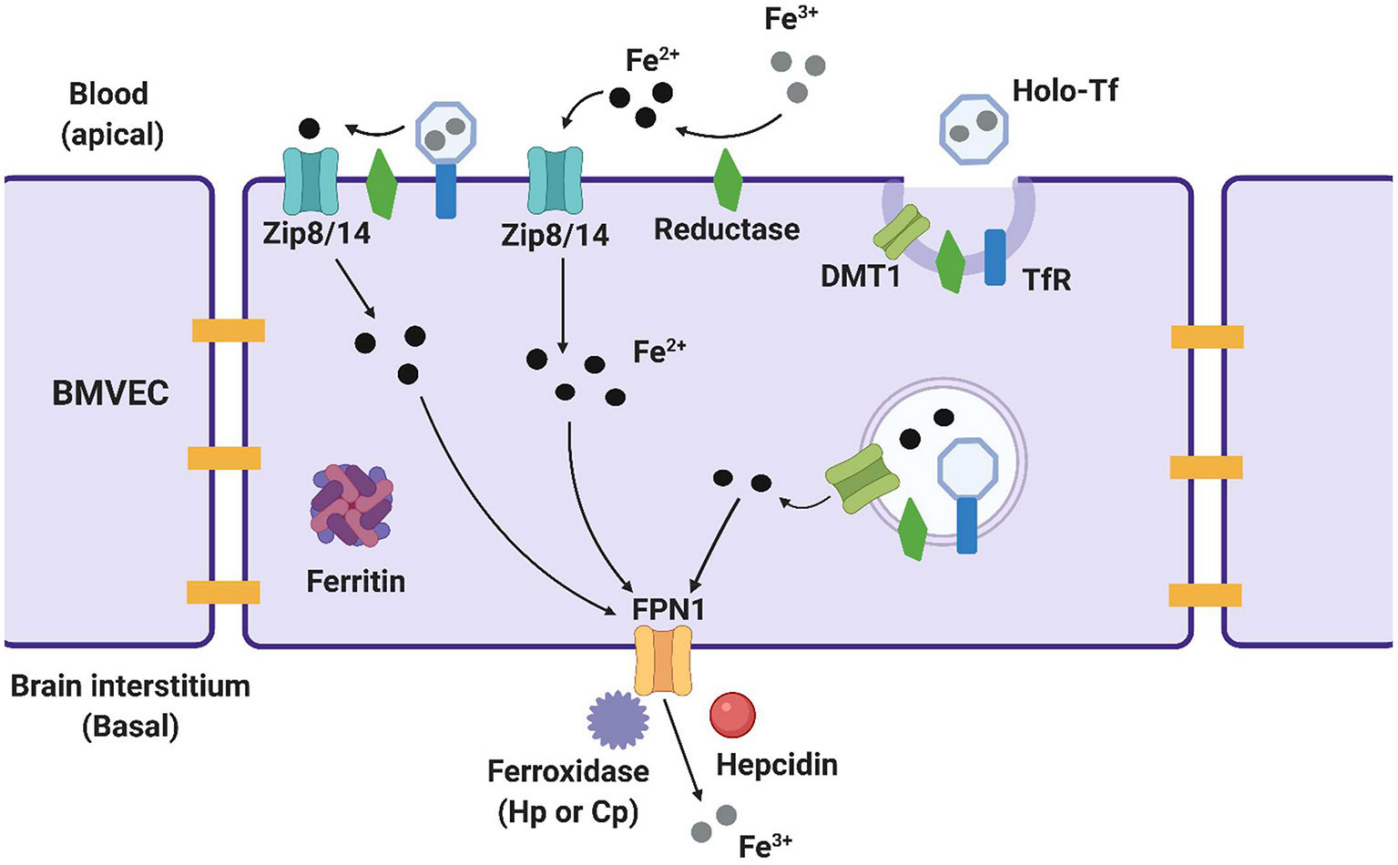
Methods of iron trafficking in BMVECs. This image highlights the uptake and efflux pathways used for iron transport in BMVECs. The yellow bars represent tight junctions which will be discussed in a later section. ZIP8/14 can transport transferrin bound iron (TBI) and non-transferrin bound iron (NTBI), with the help of a reductase which reduces the iron and allows it to enter the cell. Ferric (Fe^3+^) iron binds to transferrin, creating holo-Tf which binds to TfR and is endocytosed into an endolysosome in the cell. Here, the iron is reduced and leaves the endolysosome through DMT1. Within the cell, ferrous iron (Fe^2+^) can be stored in ferritin for later use. When the iron is ready to exit the cell, it is first oxidized back to ferric iron by a ferroxidase, and exits through ferroportin (FPN). Hepcidin is an effector hormone known to induce degradation of ferroportin and prevent cellular iron efflux. This image was created with Biorender.com.

Several proteins on the luminal, or systemic blood membrane, and on the abluminal, or membrane closest to the brain interstitium, of the BBB mediate iron uptake and efflux into the brain. Iron uptake occurs through either transferrin-bound iron (TBI) or non-transferrin bound iron (NTBI) mechanisms. The TBI uptake pathway involves the binding of iron to the transferrin (Tf) protein, the binding of transferrin to the transferrin receptor (TfR), endocytosis of this complex, and finally, iron reduction and release into the cytosol through the divalent metal transporter 1 (DMT1) (19). NTBI uptake can occur by first either, reduction of extracellular free ferric iron (Fe^3+^), or reduction of TBI by a ferrireductase, Steap2/3. Once reduced, NTBI is brought into the cell by either ZIP8 (gene *SLC39A8*) or ZIP14 (*SLC39A14*), which are divalent metal ioin transporters that also transport Zn^2+^ and Mn^2+^ (20). Once in the cytoplasm of the BMVEC, ferrous iron can be chaperoned by the iron chaperones, PCBP1/2 for storage in ferritin (as Fe^3+^) or metalation of cytosolic iron-dependent enzymes; or delivered to the mitochondria for assembly of Fe,S clusters and heme. BMVEC-accumulated iron also can be exported apically back into circulation or basolaterally released into the abluminal space. Lysosomal degradation of ferritin releases ferrous iron into the cytosol when needed, with the help of the ferrireductases Steap2/3 and Lcytb (21). Ferrous iron efflux is supported by the sole iron efflux transporter, ferroportin (FPN). FPN-dependent efflux requires the subsequent ferrous iron oxidation—ferroxidation—by a ferroxidase, either ceruloplasmin (CP) or hephaestin (HP) (22). Efflux is regulated by the abundance of FPN in the plasma membrane. The peptide hormone, hepcidin, binds to FPN and triggers its internalization and degradation (23–25). Systemically, hepcidin is released from hepatocytes; in the brain, it is released from glial cells (26, 27). These pathways are displayed in Figure 1 and the proteins of interest are presented in Table 1. In BMVECs, endosomal-independent NTBI and TBI incorporation pathways are likely to be the main mode of iron transport into the brain. Evidence for this comes from many veins, showing that removing ferrireductases, chelating extracellular iron, and knocking down the ZIP proteins all inhibit TBI uptake (20, 28). These results implicate ZIP-mediated iron transport as the most prominent method of iron accumulation in BMVEC, the first step in the accumulation of iron in the abluminal space and the glia and neurons therein. Much is understood about the basic cellular processes involved with iron metabolism, but how these processes are affected by chronic inflammation is an underrepresented area of investigation.

**Table 1:** Iron metabolism proteins in BMVEC.

**Classification**	**Protein**	**Note**	**References**
Uptake transporter	ZIP8	TBI and NTBI transport	(18, 20)
	ZIP14	TBI and NTBI transport	(18, 20)
	Transferrin (Tf)	TBI transport	(17, 20)
Efflux transporter	Ferroportin (FPN1)		(20)
	Divalent metal transporter 1 (DMT1)	Endolysosomal and lysosomal efflux	(17, 20)
Receptor	Transferrin receptor (TfR)		(17, 20)
Storage	Ferritin		(19, 20)
Enzyme	Steap 2/3	Ferrireductase, Lysosomal Ferrireductase	(19, 20)
	Lcytb	Lysosomal Ferrireductase	(19)
	Ceruloplasmin	Ferroxidase	(20)
	Hephaestin	Ferroxidase	(20)
Effectors	Hepcidin	Inhibits iron efflux through effects on ferroportin	(21–23)

## Chronic Inflammation

**Figure 2:**
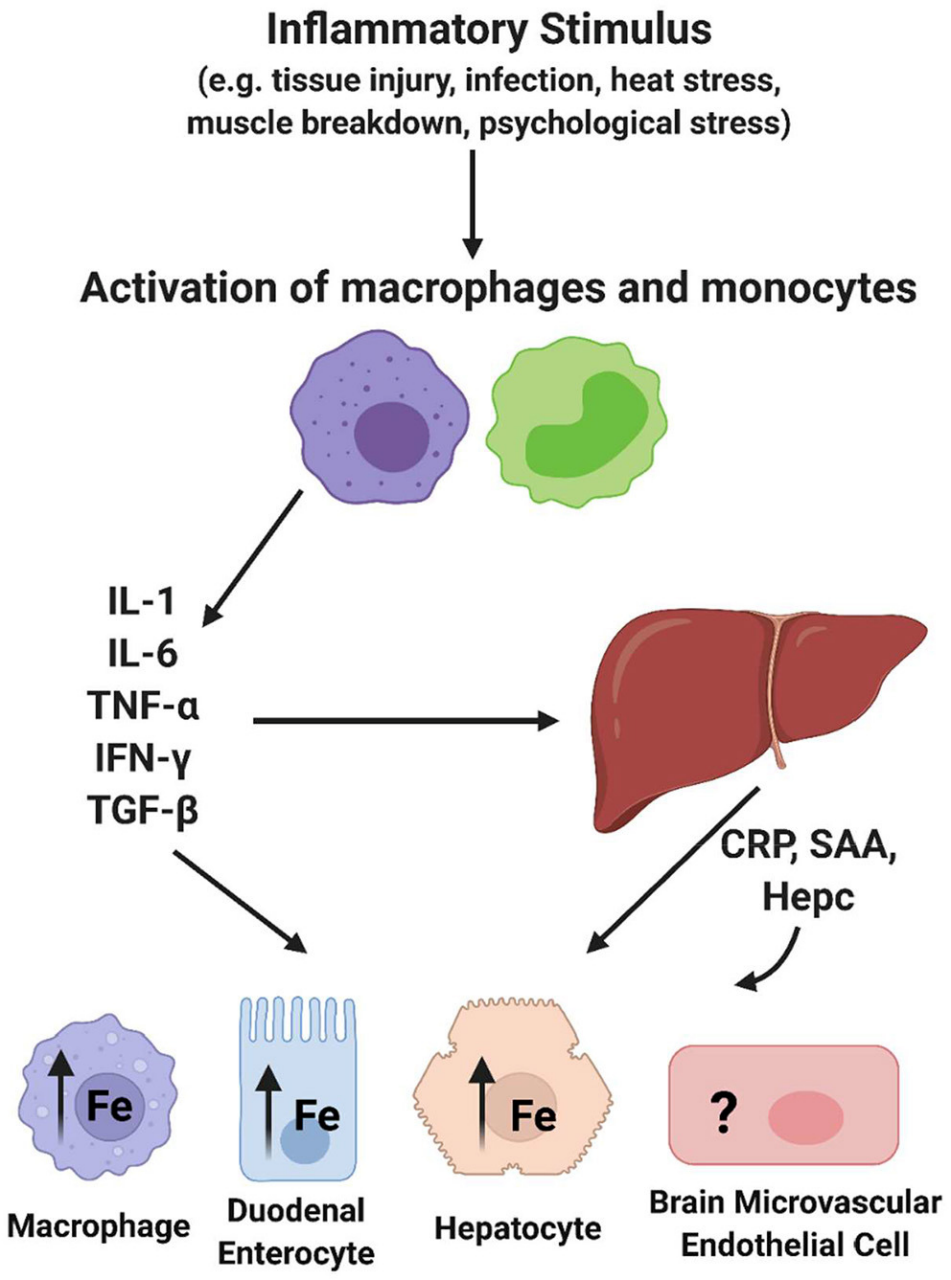
Acute phase response effect on cellular iron trafficking. Essentially all of the cytokines released upon activation of macrophages and monocytes trigger changes in most if not all tissues with impact on their iron trafficking. In addition, several of the acute phase proteins released from hepatocytes (APP) have a sustaining effect on these processes. Hepcidin (Hepc) has the specific role of modulating the steady-state level of the sole iron efflux transporter, Ferroportin (FPN), in a cell's plasma membrane, thus regulating cell iron efflux. This image was created with Biorender.com.

The focus of this review is broadly on the relationship between inflammation and iron accumulation in the brain. More specifically, it is to describe knowledge about the mechanisms behind brain iron accumulation that presents in chronic inflammatory disorders. To understand chronic inflammation, acute inflammation must be discussed. Acute inflammation can be activated by changes in body homeostasis such as bacterial infection, viral infection, body injury, or immunological disorders. These occurrences can induce the acute phase response (APR), which is characterized by changes in the concentration of plasma proteins known as acute phase proteins (APPs) (29). Following an immunological trigger, monocytes become activated and release cytokines (IL-6, TNFα, IL-1, IFN-γ, TGF-β). These cytokines induce the synthesis and release of acute phase proteins from hepatocytes in the liver (Figure 2). Of note, IL-6 is known as the most potent cytokine that induces the APR (30, 31). An acute phase protein is any plasma protein that increases or decreases by 25% during inflammation (29). Interestingly, there are several APPs that are associated with iron homeostasis. For example, concentrations of the iron storage protein ferritin and the ferroxidase ceruloplasmin increase during acute phase inflammation. Also, the iron uptake protein transferrin is decreased during this response. Once released, APPs can travel to various tissues and initiate downstream effects relating to an inflammatory response, such as fever, leukocytosis, and muscle breakdown (29). One relevant downstream effect of the APR is a decrease in the serum concentration of iron, or hypoferremia, suggesting that iron is being sequestered within cells and tissues (32). Of high importance, another APP, hepcidin, is a key regulator of this effect due to its ability to prevent iron efflux from cells (33). Indeed, a significant feature of the inflammatory response due to this hepcidin release is iron sequestration in macrophages (34–36), duodenal enterocytes affecting dietary iron absorption (37–39), and in hepatocytes (40), but the effect of inflammation on brain iron sequestration is not well understood (Figure 2). Overall, acute phase inflammation and its relationship to iron homeostasis is relevant to understanding the connection between chronic inflammation and brain iron accumulation.

Chronic inflammation is characterized as slow, long-term inflammation that lasts for several months to years. This long-term inflammation can be due to failing to remove the agent causing acute inflammation, such as a virus or bacterial infection, autoimmune disorders, such as rheumatoid arthritis, or chronic diseases that involve an inflammatory response (41). Features of acute phase inflammation continue as the inflammation becomes chronic, specifically vasodilation, increased barrier permeability, and activation of monocytes and resulting release of pro-inflammatory cytokines. This helps contribute to the goal of chronic inflammation, which is infiltration of the primary inflammatory cells into the tissue site, production of inflammatory cytokines and growth factors, and resulting tissue damage and/or repair (42). Chronic inflammatory diseases are the leading cause of mortality worldwide and therefore the biggest threat to human health (43). Highly prevalent chronic inflammatory disorders include diabetes, stroke, asthma, coronary heart disease, and allergies. Neurodegenerative disorders often present with chronic inflammation, and therefore are also considered chronic inflammatory disorders (41). Several studies have shown increased expression of pro-inflammatory cytokines in neurodegenerative disorders including Alzheimer's Disease (AD), Multiple Sclerosis (MS), and Parkinson's disease (PD) (44–48). In the brain, chronic inflammation is mediated by microglia and astrocytes, which respond to signals from the systemic circulation and subsequently produce inflammatory stimuli themselves (Table 2). Specifically, microglia, which are the macrophages of the brain, become activated when signals from systemic serum are released and cross the BBB (55). Activated microglia release inflammatory signals that activate astrocytes to a reactive phenotype, in which they release several cytokines and factors affecting the brain microvascular endothelial cells and circulating through the brain interstitium (56). This chronic inflammatory activation can perpetuate neuronal damage, neurodegeneration, and subsequent cognitive decline, of which the mechanisms are not yet fully understood. Altogether, inflammation leads to a cascade of events and the inflammatory stimuli involved, when chronic, lead to alterations in iron metabolism in the periphery, and possibly in the brain.

**Table 2:** Glial cell responses to inflammation in normal aging and neurodegenerative disorders.

**Condition**	**Glial cell**	**Response**	**References**
Normal Aging	Microglia	Increased MHC expression, complement proteins, integrins, toll-like receptors	(49, 50)
	Microglia	Release TNFα, C1q, IL-1α	(48)
	Astrocyte	A1 reactivity = secretion of toxins that kill neurons, loss of normal astrocyte functions (promotion of neuron survival, promotion of synapse growth)	(48)
Parkinson's Disease	Microglia	Increased MHC II expression, association with CD4+ and CD8+ T cells	(51, 52)
	Astrocytes	A1 reactivity	(48)
Alzheimer's Disease	Microglia	Cytokine production and release, loss of ability to clear amyloid β	(53, 54)
	Astrocytes	A1 reactivity	(48)

Consequences of chronic inflammatory mediators have mainly been studied on the endothelial cells of other known barriers, especially those of the macrovascular endothelium. For example, CRP has been shown to affect macrovascular endothelial cells by inducing cytokine release and upregulating the expression of adhesion molecules, promoting a proinflammatory response and atherosclerosis (57–59). IL-6 also activates macrovascular endothelial cells and increases adhesion molecule expression and chemokine release (60–62). Lastly, TNF cytokine family members increase adhesion molecule expression and chemokine production in macrovascular endothelial cells (63–65). Therefore, it is known that inflammatory signals associated with a chronic inflammatory response alter macrovascular endothelial cells and promote inflammation, but the function of these signals with respect to BIA mediated by BMVECs has not been elucidated.

## Normal Aging And Inflammation

With normal aging, there is physiological and cognitive deterioration. One theory to explain this has been described as the “free radical theory of aging,” basically stating that with age, there is greater production of reactive oxygen and nitrogen species, activating transcription factors and leading to the generation of pro-inflammatory molecules and a chronic inflammatory state. Developing on this theory, the chronic inflammatory state seen in aging is characterized by local infiltration of macrophages into tissue sites of inflammation, and increased levels of circulating pro-inflammatory cytokines, complement components, and adhesion molecules (66, 67). This chronic inflammatory state is especially displayed in the aged brain, which has been shown in both mouse and human studies. For example, older mice injected with LPS had an exaggerated inflammatory response indicating amplified neuroinflammation compared to young mice (68). Also, the brains of aged mice have greater levels of IL-1, IL-6, and reduced expression of the anti-inflammatory cytokines IL-10 and IL-4 (49, 69–71). Of importance, when primed with LPS, old mice had an increased inflammatory response in the hippocampus compared to younger cohorts which was accompanied by neurobehavioral complications. The same group found that hippocampal processing in these old mice was more easily disrupted than in younger ones when the peripheral innate immune system was stimulated (50). This indicates that inflammation may have negative downstream effects on cognition in aged brains. In the aging brain, glial cells respond in a pro-inflammatory manner. Microglia in the aged brain become primed to respond to inflammation (Table 2). They have greater MHC II expression, complement proteins, integrins and toll-like receptors, all allowing for a stronger pro-inflammatory reaction (72, 73). Also, microglia morphology changes with aging, as the cells become smaller with less process ramifications and less motility, allowing them to stay at inflammatory sites longer (74). Controversial questions still remain about whether aged, primed microglia have a better ability to respond to neuroinflammation or if their change in morphology is more reflective of senescence and dystrophy (75–77). Aged microglia release factors that can activate astrocytes to a reactive A1 phenotype, including TNFα, C1q, and IL1α (56). The aging induced A1-like reactive astrocyte phenotype is characterized by loss of normal astrocyte functions such as promoting neuron survival and synapse formation. Most importantly, A1 astrocytes become strongly neurotoxic and have the ability to rapidly kill neurons in the aged brain (78). These inflammatory alterations in the brain likely contribute to the neurodegeneration that takes place with normal aging.

Aging-induced inflammation contributes to another important phenomenon, anemia of inflammation. Anemia of inflammation (AI) can occur in patients with infection, sepsis, chronic inflammatory disorders, or in normal aging, in which it is called anemia of aging. As mentioned, prevalence of anemia increases with age, and is accompanied by rises in expression of pro-inflammatory markers, including CRP and IL-6 (79, 80). AI is characterized by decreased serum iron concentrations despite normal amounts of the iron storage protein ferritin, representing iron stores. Alterations in the expression of acute phase proteins is a defining feature of AI. First, the acute phase protein hepcidin plays a highly important role in this anemia. As mentioned previously, in an inflammatory state, there are greater hepcidin levels, which inhibits iron efflux by effects on the iron efflux protein ferroportin. A hallmark of AI is hepcidin-mediated hypoferremia that is induced by increased expression of cytokines. The concentration of serum transferrin, another acute phase protein, decreases in AI (81). How this affects brain iron accumulation is unknown, but a lack of transferrin would likely induce an increase in ZIP-mediated iron uptake, although more research is needed to confirm this. Overall, much is understood about systemic changes that occur with anemia of inflammation and aging, but little is known about what happens with respect to iron uptake and accumulation in the brain.

## Normal Aging And Brain Iron Accumulation

Unbalanced brain iron concentrations can result in neuronal death and subsequently, a loss of cognitive and motor function. Iron concentrations increase during the aging process in different human brain regions. As determined with MRI methodology, this iron is most strongly concentrated in areas of the basal ganglia, specifically in the substantia nigra and the striatum (82) (Table 3). In the substantia nigra, increases in different molecular forms of iron are found with aging, including total iron, heavy and light chain ferritin, and the neuromelanin-iron complex (16, 83, 103). In mice, similarly to humans, age-associated increases in iron particularly in the white matter tracts of basal ganglia regions were found. This was accompanied by glia dystrophy, removing the ability of the astrocytes and microglia to sequester iron and prevent neurotoxicity (104).

**Table 3:** Evidence of brain iron accumulation in aging and neurodegenerative disorders.

**Condition**	**Species**	**Methods**	**Result**	**References**
normal aging	Human	MRI	Iron most strongly concentrated in areas of the basal ganglia, specifically in the substantia nigra and the striatum	(80)
	Mouse	Immunohistochemistry, Elemental mapping	Increases in iron in white matter tracts of basal ganglia	(81)
	Rat		Greater substantia nigra iron content, increased ferritin, lipid peroxidation	(83)
	Rat	Immunocytochemistry, Western blot	Increased HO-1 expression in areas known for aging-induced brain iron accumulation	(84)
Alzheimer's Disease	Human	Postmortem histochemistry	Increased iron content in basal ganglia, greater ferritin, decreased transferrin	(85)
	Human	Postmortem histochemistry	Increased redox-active iron associated with senile plaques and neurofibrillary tangles	(86, 87)
	Human	SWI MRI	Increased brain and body iron levels	(88)
	Mouse	APP/PS1 mouse model, X-ray fluorescence microscopy	Iron associated with amyloid β plaques	(89, 90)
Multiple sclerosis	Human	Postmortem histochemistry	Increased iron in white and gray matter areas	(91, 92)
	Human	QSM-MRI	Higher susceptibility suggesting increased iron in basal ganglia regions	(93)
	Mouse	EAE mouse model, MRI, Histochemistry	increased brain iron content, iron deposits found near areas of demyelination and activated microglia	(94–96)
Parkinson's Disease	Human	Postmortem histochemistry	Higher iron content in substantia nigra associated with microglia and dopamine neurons	(97–100)
	Human	QSM-MRI	Greater iron deposition in substantia nigra	(101)
	Rat	6-OHDA treatment, histochemistry, immunohistochemistry	Greater iron in substantia nigra, decreased ceruloplasmin expression	(102)

Studies focused on the accumulation of iron in neuromelanin-containing organelles have provided needed analytical evidence of the co-morbidity of brain iron accumulation in normal aging and associated with neurodegeneration. Using analytical transmission electron microscopy and nano-secondary ion mass spectroscopy, Biesemeier et al. demonstrated that iron was present at ~0.15 mol percent in these organelles, three-fold greater than for copper, for example (84). Neuromelanin has promise as a bio-marker of Parkinson's disease (105) and given the association of iron with this autophagic marker of catecholamine oxidation, it can be captured by MRI. However, one difficulty moving forward is to distinguish between the normal increase in neuromelanin with age and a co-morbid change associated with disease on-set (106). While not useful for the detection or treatment of disease, there are two new approaches that can provide higher resolution in the effort to localize the sites of brain iron accumulation. One of these is Scanning Transmission X-ray Microscopy (STXM). In this technique, at a given energy, the absorbed photon knocks an element's 1s electron into the continuum; for iron, this is known as the K-edge, and it differs for ferrous (Fe^2+^) in comparison to ferric (Fe^3+^) (107). Furthermore, given the plane polarized nature of light—including X-rays—the ‘optical activity' in the iron's electronic structure can be exposed. In this way, Telling et al. could dissect the redox state and differential coordination chemistry of iron in amyloid plaques in a mouse model of Alzheimer's Disease (108) and in post-mortem tissue from Alzheimer's Disease patients (107). Another developing technique for the high-resolution (~1–2 μm) quantification of metal ions is *micro*Particle Induced X-ray Emission (μPIXE) (109). Rather than an X-*photon* as in STXM, μPIXE uses a proton (the “particle”) to couple with an energy transition in the metal ion. Using this approach, Friedrich et al. showed that in mid-brain (substantia nigra) sections from controls, oligodendroglial and astroglial cells were iron-rich, whereas in sections from Parkinson's disease patients, iron was increased throughout *except* for these two cell types (110). In addition, this neuronal iron increase appeared to be localized in the cytosol indicating an increase in the labile iron pool. An obvious extension of these experiments is the comparison between the *quantitative* neuronal iron increase revealed by the μPIXE approach and delineation of the redox state and coordination derived from the STXM measurement.

A question of how increased iron accumulates in the aging brain still persists. In aged mice, increased expression of the iron transporter DMT1 have been found (51, 111). Interestingly, in aged mice, expression of the transferrin receptor decreases (51). Decreases in the proteins of the transferrin uptake pathway take place in both aging and inflammation, suggesting again that ZIP-mediated transport may be more important for iron accumulation in these states. One noteworthy study injected young and aged rats with LPS and measured the amount of iron in the substantia nigra, the brain region most affected by Parkinson's Disease. Aged mice had greater substantia nigra iron content, along with increased ferritin expression, microglia activation, and lipid peroxidation (52). This study shows that inflammation may play a role in the brain iron accumulation that is seen in aging. Expression of heme oxygenase 1 (HO-1) increases with age. HO-1 is an enzyme that degrades heme, releasing several molecules including carbon monoxide and ferrous iron (Fe^2+^) (53, 54). Higher levels of systemic inflammation in aging can increase the expression of HO-1 in the brain, an increase that is co-morbid with cognitive impairment (112). Therefore, upregulation of HO-1 may be a component of brain iron accumulation with inflammation that is seen in aged populations. Another factor that can contribute to brain iron accumulation with aging is increased expression of abluminal hepcidin. In aged mice, hepcidin increases in the cortex. This results in decreased surface expression of ferroportin, and an increased cytosolic non-heme iron that would result from the expected knock down of ferrous iron efflux (113). This may result in iron accumulation in any of the various cell types encompassing the cortex. Therefore, increased expression of hepcidin may contribute to a greater brain iron retention associated with aging. This retention would predict a decrease in efflux of iron from the brain, into either the cerebral-spinal fluid, or by an abluminal to systemic flux of iron *via* the microcapillary endothelium of the blood brain barrier. Thus, the mechanisms for age-related increases of iron content are not clear, but clearly relevant to the accumulation of dysregulated iron in the aging brain.

## Neurodegenerative Disorders And Inflammation

Unhealthy aging, as in neurodegenerative disorder presentation, is accompanied by elements of an inflammatory response. As stated earlier, cytokine expression is upregulated in neurodegenerative disorders. For example, CSF collected from MS patients showed greater expression of IL-6 and TNFα compared to patients with non-inflammatory neurological diseases (114). Also, evidence of an abundance of macrophages and CD3+ T cells in MS brain tissue samples, including in patients with progressive disease, indicates continuous activation of the innate and adaptive immune system throughout prolongation of the disease (115). Systematic meta-analysis of several AD patient CSF cytokine studies revealed heightened expression of TGF-β and MCP-1 in AD patients compared to controls, suggesting that these cytokines could be used as biomarkers for this disease (85). Studies have found increased levels of pro-inflammatory cytokines, such as TNFα, IL-1β, and IL-6, in the nigrostriatal region of postmortem brains and in the CSF of patients with PD (86, 87). Serum samples taken from PD patients display amplified levels of monocytes, neutrophils, leukocytes, and CRP expression, suggesting the presentation of a heightened inflammatory response (116). Inflammatory mediator expression is a well known aspect of neurodegenerative disease and may be an upstream explanation for neurodegeneration and cognitive decline.

Glial cell activation and perpetuation of inflammatory response is prominent in neurodegenerative disorders. In the postmortem substantia nigra of PD patients, significant microglial activation has been found, correlating to increased MHC-II expression (91) (Table 2). MHC-II expressing microglia were discovered in the substantia nigra of a rat model of PD, along with CD4+ and CD8+ T cells, suggesting these microglia are in an environment where they are primed to present Parkinson's related antigens for T cell recognition, furthering disease pathogenesis (92). Microglia activation, release of cytokines, and induction of neuron death is associated with AD pathology. Aβ production and failure to clear this protein are key components in the development of AD. As this disease progresses, persistent microglia activation and release of cytokines and ROS increases Aβ generation and decreases clearance of this harmful protein (97, 117). These examples of glial cell mediated inflammation advocate for the idea that glial cells may play a prominent role describing mechanisms of inflammation induced brain iron accumulation.

Several studies with mice have made clear that inflammation can have an important effect on neurodegenerative disease pathogenesis. In a study examining AD, treating wild type ICR mice with LPS resulted in memory impairment as shown by the passive avoidance and the water maze tests. These cognitive deficits were accompanied by Aβ generation in the cortex and hippocampus and greater neuron death when treating with LPS (98). Similar results were seen when giving wild type C57BL/6J mice LPS for a longer period of time, confirming that long-term chronic inflammation can perpetuate AD pathophysiology and cognitive impairment (99). Another study investigating how systemic inflammation can result in neurodegeneration found that one systemic injection of LPS into adult wild type mice can produce increased levels of brain TNFα for 10 months. Researchers in this group found that this increase in brain TNFα was accompanied by activated microglia in the cortex, hippocampus, and substantia nigra. Lastly, they discovered a loss of dopamine neurons that did not become apparent until 7 months after the initial LPS treatment (100). This study illustrates that sustained neuroinflammation induced by a systemic stimulus can be a source of harm to neurons and can attenuate PD disease progression. Overall, several lines of evidence state that neurodegenerative disorders present with systemic and neuroinflammation, but mechanisms describing the effects of inflammation on disease progression are still lacking and need continued research.

## Neurodegenerative Disorders And Brain Iron Accumulation

One possible pathway to the neurodegeneration seen in neurodegenerative disorders is increased brain iron accumulation (Table 3). Increased disordered iron in brain tissue can result in greater production of ROS and neuronal death. Research in this area has been conducted using human postmortem tissue samples, human brain MRI, and animal models. Elevated iron levels in postmortem AD brains was first discovered in 1953 (88). Several studies following confirmed this, and added that iron colocalizes with the senile Aβ plaques and neurofibrillary tangles associated with this disorder (93, 118–120). Histopathological studies on MS patients have also been completed, finding increased brain iron accumulation in both gray and white matter regions (101, 121). One specific study found iron in normal controls near oligodendrocytes and myelin fibers, but when demyelination in MS patient lesions was analyzed, iron was more closely associated with microglia, suggesting dysregulation of iron homeostasis in this disease (94). In postmortem PD tissue, increased iron levels are found in the substantia nigra. Specifically, this iron has been detected associating with microglia and dopaminergic neurons, signifying iron deposition in these areas as a contributor to the neuronal death seen in this disease (95, 96, 122, 123). Excess reactive iron becomes associated with neuromelanin within neurons and extracellularly. When these neurons die, neuromelanin remains in the extra neuronal space and stimulates microglial release of neurotoxic factors including TNF-a, IL-6, and nitric oxide. This microglial activation is also stimulated by Aβ-iron complexes, suggesting a pattern of protein aggregation, metal complexing, and induction of inflammation (124). This release of inflammatory factors may function to increase brain iron accumulation through effects on iron transporters and BBB permeability, ultimately aggravating the neuropathology.

fMRI methods have been widely used to confirm alterations in iron concentrations of patients with neurodegenerative disorders. Iron, in general, is the most abundant paramagnetic ion in the brain. Different forms of iron in the brain have varying levels of paramagnetism, altering their magnetic susceptibility, or response to an applied magnetic field. For example, ferric iron stored in ferritin, a main source of iron in the brain, is superparamagnetic. In contrast, although oligodendrocyte-dependent myelin biosynthesis is strongly iron-dependent, much of this iron is the ferrous form and is diamagnetic. Thus, white matter areas of the brain display both dia- and para-magnetism. One important fact to consider is that MRI methods do not yet have a way to distinguish different molecular forms of iron, an area needing further development (102). Another approach is susceptibility weighted imaging. SWI is an MRI technique that uses the paramagnetic property of the iron storage protein ferritin to measure a phase shift relating to the amount of iron in the tissue, has been used to investigate iron in the brains of AD, PD, and MS patients. In all disorders, SWI revealed increased iron content in several brain regions compared to healthy patients (89, 90, 125, 126). QSM-MRI, a more advanced and quantitative method used to measure brain iron *in vivo*, measures the susceptibility of tissues to paramagnetic species. As noted, many forms of iron are paramagnetic and therefore this MRI method has been accepted as a quantitative method to assess tissue iron levels including iron content in neurodegenerative disordered brains. For example, QSM-MRI has demonstrated increased iron content in MS, PD, and AD brains (127–129).

Animal models of neurodegenerative disorders have been utilized to determine the involvement of altered brain iron metabolism in disease progression. Similar to humans, a mouse model of MS called experimental autoimmune encephalomyelitis (EAE), displays increased brain iron content, measured by both MRI and histochemical techniques. These iron deposits were found near areas of demyelination and activated microglia (130–132). This highlights a possible mechanism for iron accumulation and neurodegeneration in MS, which is that oligodendrocyte death leads to myelin destruction, iron release from dying oligodendrocytes, subsequent oxidative damage and neuron death. Injection with 6-hydroxydopamine (6-OHDA) is a method commonly used to create a mouse model of PD. Using this model, techniques such as elemental bio-imaging, immunohistochemistry, and MRI have been implemented to measure iron levels. Several pieces of evidence from these studies reveals increased iron content specifically in the substantia Nigra in the brains of animals modeling PD (133–135). In this same model, decreases in the iron efflux protein ferroportin and the ferroxidases hephaestin and ceruloplasmin were discovered, suggesting iron sequestration in CNS cells as a mechanism for dysregulated iron homeostasis in PD (136, 137). Lastly, the APP/PS1 mouse model of AD, which displays amyloid plaques closely resembling those of human AD, has been utilized to measure iron accumulation with neurodegeneration. Using this model, increases in microglial-iron retention and iron surrounding Aβ plaques was determined (138, 139). Altogether, there is ample research providing evidence for a relationship between brain iron accumulation and neurodegenerative disorders. Mechanisms for what triggers this iron accumulation need ongoing study to fully understand and prevent neurodegenerative disease propagation.

## Mechanisms For Brain Iron Accumulation With Inflammation

Much is known separately about the processes of inflammation and iron metabolism in healthy aging and neurodegenerative disease progression. What is still unclear is whether there is a cause and effect relationship between these two phenomena that can explain the cognitive decline that is present in both healthy and diseased aging brains. Considering the information presented above, two mechanisms explaining how brain iron accumulation occurs in states of inflammation will be discussed.

### Inflammation and Iron Transporters: Transcellular Brain Iron Accumulation

**Figure 3:**
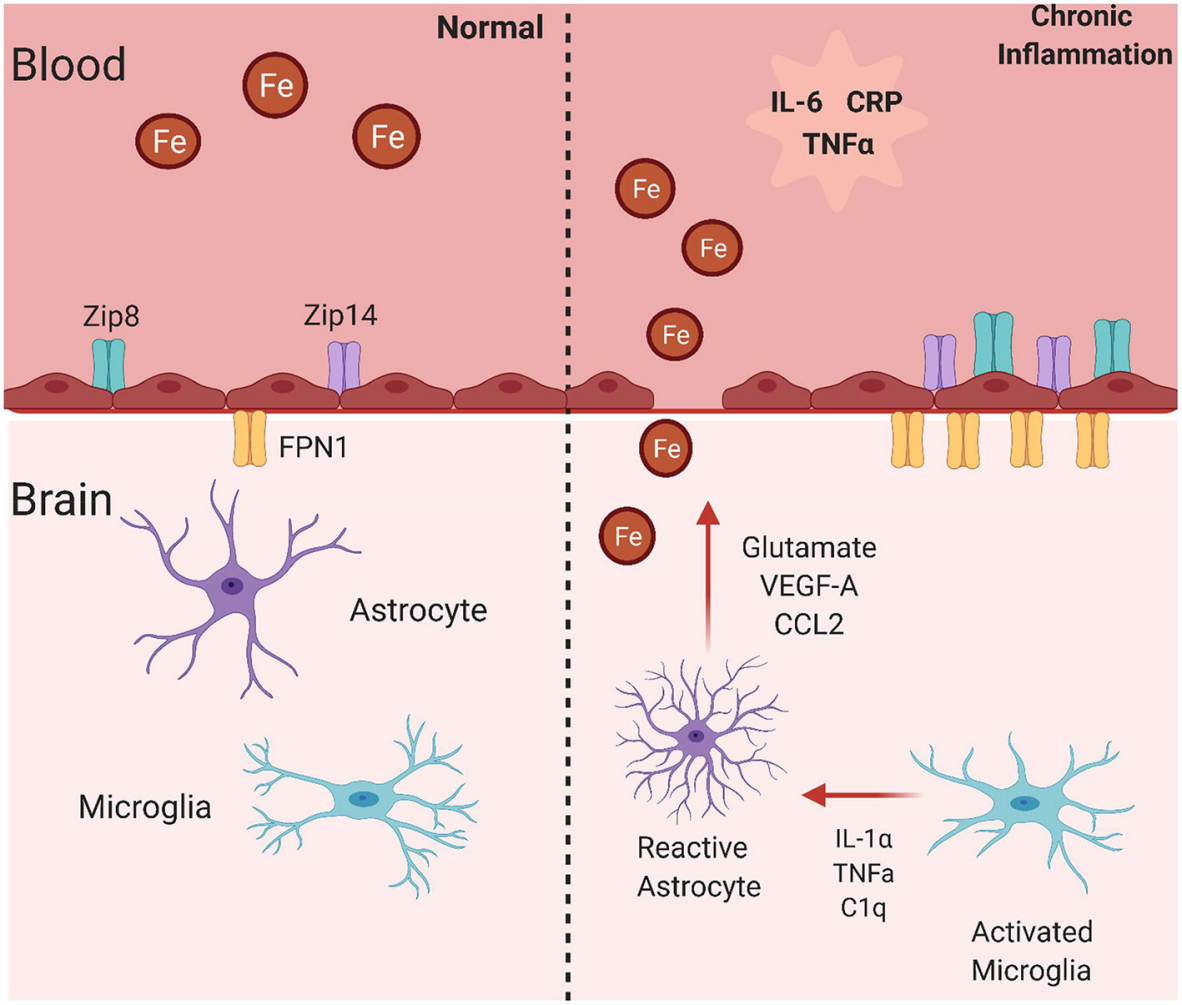
Mechanisms for brain iron accumulation in a state of chronic inflammation. In a state of chronic inflammation, systemic inflammatory factors increase plasma membrane iron transporter occupancy. These include the ferrous iron uptake transporters, ZIP8 and ZIP14, and the sole iron efflux transporter Fpn. Note that these changes occur at both the apical (blood) and basolateral (brain) side of the endothelial cell blood-brain barrier in response to systemic and abluminal signals. This image was created with Biorender.com.

A likely mechanism for increased BIA in an inflammatory state is functional changes in iron uptake and efflux proteins (Figure 3). Specific inflammatory mediators are known to upregulate expression of iron metabolism related proteins. Also, a noteworthy study revealed that treating hippocampal neurons, cortical astrocytes, and cortical microglia with IL-6, TNFα, and LPS increases expression of DMT1 and decreases expression of Fpn1. The changes in these proteins was correlated to greater iron uptake into the hippocampal neurons and cortical microglia (140). Transgenic mice expressing IL-6 in astrocytes present with increased iron deposition in the cerebellum, greater ferritin expression, and decreased transferrin expression (141). Lastly, microglia are known to take up NTBI in response to LPS, which occurs through increased DMT1 expression (142). This research demonstrates that inflammatory mediators have the ability to alter iron-related protein synthesis and resulting iron accumulation, although the mechanisms behind this are not known. The decrease in FPN expression in response to inflammation begs the question of how would abluminal iron efflux from BMVECs occur. Stated before, inflammation induced hepcidin would likely decrease expression of FPN in BMVECs. One study found that mouse astrocytes overexpressing hepcidin reduced BMVEC expression of TfR1 and FPN, suggesting reduced iron flux across the BBB (143). Although this study lacks functional determination of iron flux across BMVEC and determination of non-canonical iron accumulation pathways, it highlights the ability of hepcidin to affect proteins involved with iron accumulation. Another report provided further insight, showing that glia grown in contact with BMVEC released hepcidin that knocked down BMVEC FPN-dependent iron efflux (144). Certainly, as inflammation is known to increase hepcidin, more research is needed to determine if inflammation increases or decreases iron flux across the BBB. While this information is provided in these cell types, research into how IL-6, and other significant inflammatory signals, affect iron uptake transporters in BMVECs is lacking. As mentioned previously, brain iron accumulation must occur at the BMVECs of the BBB, therefore it is extremely essential to understand how iron transporter trafficking is affected in this cell type.

As noted throughout this article, iron uptake into BMVECs is mostly mediated through the ZIP proteins, which stands for zinc-regulated, iron-regulated transport proteins (Figure 3). The ZIP protein family is very large, but ZIP8 and ZIP14 are the most homologous with 50% amino acid similarity (145). Structurally, ZIP proteins have 8 transmembrane domains, extracellular N- and C-termini, and 4 intracellular loops. The metal binding site is within transmembrane domain 5 and is conserved as HEXPHEXGD, except for in ZIP8 and ZIP14 in which the first histidine is changed to a glutamic acid. This is predicted to broaden the metal ion substrate range to include iron, zinc, manganese, and cadmium, unlike other ZIP proteins (145). Both of these proteins are expressed (145, 146). Both ZIP8 and ZIP14 are expressed in BMVEC, as shown by qPCR and immunologic analyses. Data from the same group demonstrated that LPS induces localization of both ZIP8 and ZIP14 to the BMVEC cell surface plasma membrane, showing that inflammation can alter localization of iron transporters (147). As LPS induces downstream cytokine release, it is likely that cytokines such as IL-6 and TNFα have similar effects on these proteins in BMVECs, but this hypothesis has not been tested. Research from multiple groups has revealed that IL-6 can increase expression of the both uptake proteins in liver hepatocytes and SH-SY5Y cells, a neuronal cell line (148, 149). Also, ZIP14 is known as the primary transporter facilitating zinc uptake in hepatocytes, which is regulated by the cytokine IL-1β (150). It has also been discovered that LPS treatment increases ZIP14 mRNA and protein expression, which was correlated to decreased serum zinc and increased liver zinc levels (151). As serum hypozincemia and hypoferremia both occur with inflammation, it is possible that inflammation is leading to ZIP14-mediated brain iron accumulation, as in the liver. Most research on inflammation and the ZIP proteins focuses on ZIP14 because of its direct relationship to IL-6, but ZIP8 can also be affected by inflammatory stimuli. ZIP8 transcript upregulation and amplified zinc uptake occurs in response to TNFα treatment in primary human lung epithelia and human immortalized lung epithelial cells (152). Treating synovial fibroblasts with TNFα and IL-1β induced higher zinc concentrations and greater expression of both ZIP8 and ZIP14, which was determined to be dependent on NF-κB signaling downstream of HIF-1α activation (153). ZIP8 expression is induced by the cytokine, IFN-γ, in intestinal epithelial cells, further propagating the changes in metal homeostasis seen in Crohn's Disease (154). This accumulation of evidence relates inflammation and the ZIP proteins to processes in numerous cell types, tissues, and diseases. This suggests that inflammation may regulate the ZIP proteins in the context of the brain, allowing for greater brain iron acquisition, oxidative stress, neuronal damage, and ultimately cognitive decline.

### Inflammation and Blood Brain Barrier Permeability: Paracellular Brain Iron Accumulation

**Figure 4:**
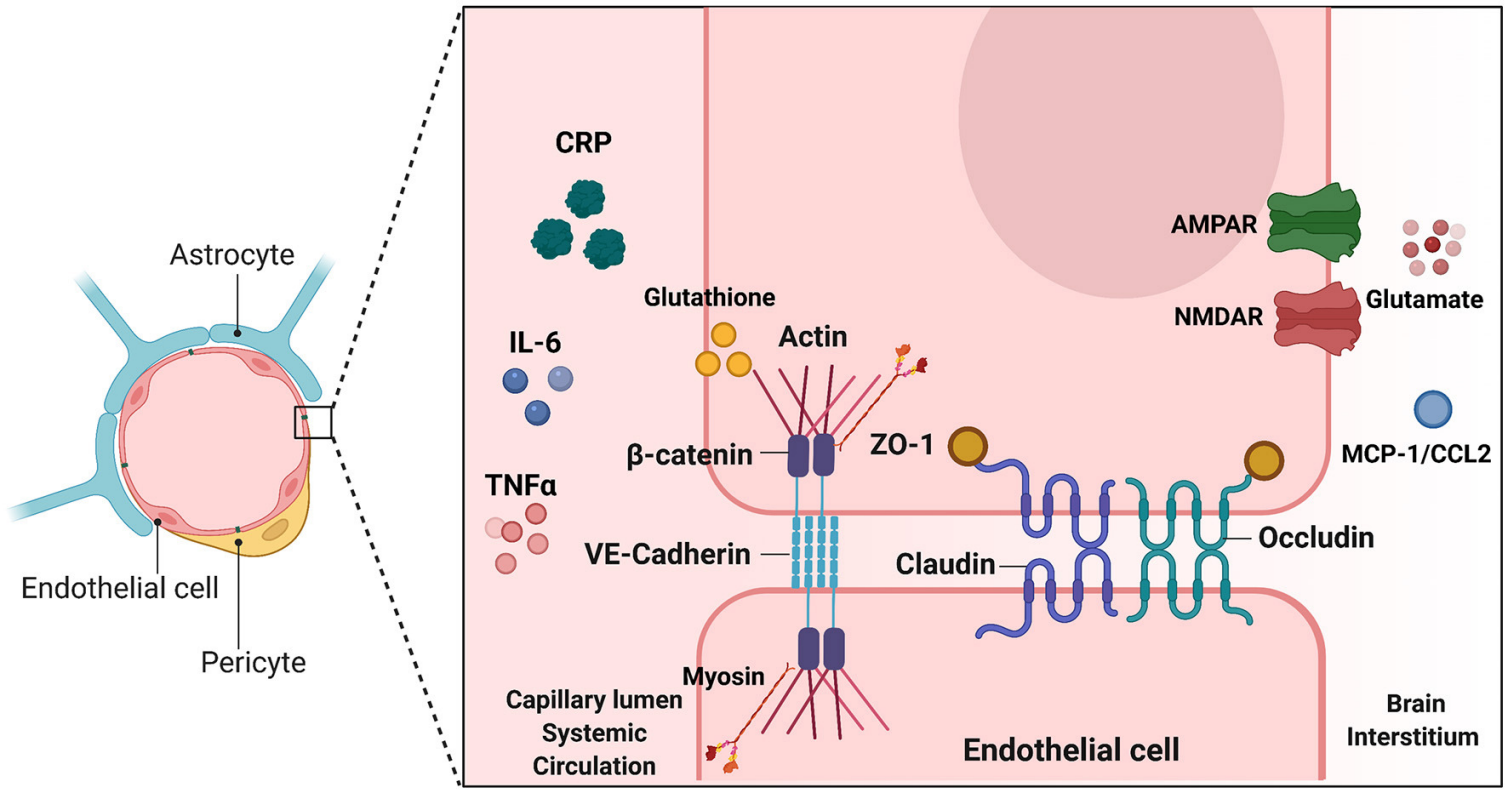
Endothelial junctions at the Blood Brain Barrier. Illustration depicting tight junctions and adherens junctions in brain microvascular endothelial cells of the BBB. Inducers of BBB breakdown are shown both in the systemic circulation and brain interstitium. TJ and AJ proteins are depicted between the two endothelial cells. Inducers shown on either side of the BBB make modifications such as phosphorylation, or glutathionylation, that have downstream effects on TJ/AJ localization and expression. This alters BBB integrity and barrier leakiness. This image was created with Biorender.com.

Changes in BBB permeability in response to inflammatory stimuli may be a significant mechanism describing BIA with inflammation (Figure 3). Permeability of the BBB refers to how well materials are able to cross, with increased permeability meaning that the barrier is less intact and more accessible to substance transport. Reviewed earlier, the BBB is composed of a neurovascular unit encompassing BMVECs, astrocytes, microglia, and neurons. Barrier permeability is controlled by tight junctions (TJ) and adherens junctions (AJ) which form between the BMVECs of the BBB. When these junctions are strong and intact, the brain's protection from systemic toxins is increased, but when they are broken down the brain is exposed to materials from the rest of the body. TJ and AJ of the cellular cytoskeleton are composed of multiple proteins converging to control permeability. TJ proteins highly expressed in BMVECs comprise claudin-3, claudin-5, occludin, and zona occludens 1 (ZO-1). AJ proteins expressed in BMVECs include VE-cadherin and β-catenin (155). Organization of these junctions in BMVECs is shown in Figure 4. Junctional proteins in many different cell types can be greatly affected by inflammation and various disease states. IL-6 receptor trans-signaling in human umbilical vein endothelial cells (HUVECs) results in a loss of ZO-1 and VE-cadherin localization, determined to be induced by STAT phosphorylation of these proteins (156). IL-6 incubated cerebral capillary endothelial cells (cEND) express less claudin-5, occludin, and VE-cadherin, and have reduced barrier functionality (157). Evidence demonstrates that the acute phase protein CRP plays a role in barrier breakdown. CRP disrupted TJ rearrangement and function of a co culture BBB model by activating a kinase that phosphorylates the myosin-light chain (158) (Table 4). Also, the blood retinal barrier (BRB) was disrupted by CRP, due to reduced expression of ZO-1 and occludin in retinal pigment epithelium (159). Oxidative stress and ROS can lead to BBB breakdown, as actin is glutathionylated in this cellular state. Actin glutathionylation limits the rate and extent of actin polymerization, in turn affecting TJ protein arrangement (160, 161). BMVECs treated with TNFα express less VE-Cadherin, occludin, and claudin-5. This correlated to increased expression of NADPH-oxidase subunits, which was shown to be responsible for the loss of TJ proteins and highlights the effect of oxidative stress on BBB integrity (162). Lastly, synergistic effects of both IL-6 and TNFα can affect BBB through decreased expression of ZO-1, claudin-5, occludin, and VE-Cadherin (163). Evidence presented above establishes that systemic inflammatory factors and oxidative stress can induce endothelial cell barrier breakdown, but factors from within the brain can have a similar effect.

**Table 4:** Cytoskeletal modifications leading to changes in TJ/AJ function and barrier permeability.

**Inducer**	**Modification**	**Result**	**References**
CRP	Phosphorylation of myosin light chain	Decrease in barrier resistance ZO-1 and occludin rearrangement	(142)
Glutathione, Oxidative Stress	Actin glutathionylation	Disrupted actin polymerization Altered TJ protein anchorage	(144, 145)
Glutamate, NMDAR and AMPAR activation	Occludin phosphorylation and dephosphorylation	Occludin rearrangement Increase in barrier leakiness	(149)
MCP-1/CCL2	TJ and myosin light chain phosphorylation by RhoA kinase	Rearrangement of ZO-1, claudin-5, occluding Disrupted actin assembly	(150, 151)
IL-6	JAK/STAT phosphorylation of ZO-1 and VE-Cadherin	Loss of ZO-1 and VE-Cadherin localization	(140)
TNFα	NADPH Oxidase-Dependent ROS Generation	Reduced expression of VE-Cadherin, occludin, claudin-5	(146)

Reactive astrocytes release factors that can have harmful effects on the BBB. In normal conditions, astrocytes take up glutamate from BMVECs, but in an inflammatory state, this function is disrupted and instead they release glutamate (164). Activation of both the NMDA and AMPA receptors is involved in glutamate-induced BBB breakdown. When glutamate binds to the NMDA receptor on BMVECs, Src kinases are activated, which phosphorylate tyrosine residues of occludin. When glutamate binds to the AMPA receptor, a PKCη phosphatase is activated which dephosphorylates serine and threonine residues of occludin. In both cases, occludin is removed from normal positioning, allowing for greater barrier leakiness (165). CCL2, or MCP-1, a chemokine released by astrocytes in inflammatory conditions, facilitates breakdown of TJ and migration of leukocytes across the BBB through changes in expression and localization of ZO-1, claudin-5, and occludin (166, 167). Astrocyte-derived vascular endothelial growth factor A (VEGF-A) breaks down the BBB by targeting claudin-5 and occludin, discovered using VEGF-A KO astrocytes (168). Overall, signals coming from both the systemic circulation and the brain interstitium can alter BBB integrity (Table 4).

The BBB is compromised in many diseases of neuroinflammation. In inflammatory diseases, a more permeable barrier would be harmful because macrophages and T cells harboring antigens to the disease would more easily pass through into the brain. This, in turn, activates glial cells, induces cytokine release, and propagates neuroinflammation. In HIV encephalitis, a complication of HIV associated with cognitive dysfunction, BBB integrity is lost. In this disease, this allows for accumulation of activated, HIV-infected macrophages and induction of reactive astrocytes, the mechanism behind this being significant loss of the TJ proteins occludin and ZO-1 (169). Systemic inflammatory pain rat models, including injection of formalin, carrageenan, and complete Freund's adjuvant (CFA) display increased BBB permeability. In both the carrageenan and CFA injected rats, this was due to decreased expression of ZO-1. Carrageenan and CFA inflammatory pain models are considered longer-term than the formalin model, suggesting that BBB breakdown is induced with chronic inflammation (170). Loss of BBB integrity is an integral aspect of neurodegenerative disorders. In EAE mice, BBB breakdown increases with disease severity, encompassing disruption of ZO-1 and actin in the spinal cord and BMVECs of the BBB (171). Results of this study correlate to what has been discovered in humans with MS, demonstrating significant BBB defects in this disease (172–174). BBB abnormalities have also been discovered in AD cases. Leakage of plasma proteins into the brain that associate with senile plaques was found in postmortem AD brains, indicating increased BBB permeability (175, 176). Also, an MRI technique called dynamic contrast-enhanced MRI (DCE-MRI), found increased extravasation of a gadolinium bolus into the CSF of AD patients compared to controls, again indicating BBB breakdown in this disease (177). Overall, many lines of evidence confirm alterations in the BBB protein architecture in inflammatory conditions and suggest a relationship between inflammation, BBB integrity, and cognitive decline. What has not been examined is if these changes in BBB permeability can lead to increased brain iron accumulation. This finding would link the inflammation presented in neurodegenerative disorders to brain iron accumulation, downstream oxidative stress, and cognitive decline, an important connection to be made.

## Therapeutic Interventions For Inflammation-Induced Brain Iron Accumulation

Several different approaches can be taken to treat BIA induced by inflammation. Targets include oxidative stress, iron itself, and inflammatory mediators. Antioxidants have shown promise as therapeutics for the oxidative stress presented in healthy aging and neurodegenerative disease. For example, N-acetyl cysteine (NAC), a precursor to cysteine known to amplify glutathione levels, increased the levels of antioxidants, decreased the amount of prooxidants, and decreased expression of IL-6, TNFα, and IL1β in aging rat brains (178). Long term oral administration of NAC markedly reduced dopaminergic neuronal loss, oxidative stress, and motor abnormalities in a mouse model of PD (179, 180). N-acetyl cysteine had similar effects in human patients with PD, increasing dopamine transporter binding as a biomarker for PD pathology (181). This result demonstrates that antioxidants have therapeutic qualities to prevent the oxidative stress seen with neurodegeneration. N-acetyl cysteine was also discovered to prevent manganese toxicity in SH-SY5Y cells, a dopaminergic cell line, indicating a direct effect of antioxidants on metal toxicity (182). Non-steroidal anti-inflammatory drugs (NSAID) are another possible therapeutic intervention for inflammation-induced BIA. Ibuprofen treatment led to a reduction of plaque-associated microglia and a corresponding attenuation in proinflammatory cytokine levels in brains of Tg2576 mice overexpressing human amyloid precursor protein (APP), a mouse model of AD (183). A conjugate of glutathione and Ibuprofen attenuated AD characteristics, specifically loss of Aβ, in a rat model of AD, suggesting that antioxidant and NSAID together can be used to increase efficacy in neuroprotection (184).

In addition to these off-the-shelf small molecule anti-oxidants are ones designed based on them, *eg*, idebenone, a congener of ubiquinone or co-enzyme Q (CoQ) (185–187). These are quinone-based molecules with redox properties comparable to ascorbic acid and vitamin C. Although CoQ and idebenone have exhibited some therapeutic promise, eg., as in a Phase III clinical trial of the latter in the treatment of Duchenne muscular dystrophy (188), there are no approvals for use although in some European countries it is available in special cases (189). With respect to the use of idebenone in the treatment of cognitive decline, the results are unclear; an earlier study failed to find any benefit in the treatment of Alzheimer's disease (AD) (190) while a more recent one, employing an idebenone nanorod formulation taken orally, demonstrated efficacy in a mouse model of AD (191). This result indicates that as with any potential therapeutic, efficacy is often more a matter of delivery than function at the cell level.

Iron chelation could be useful to prevent the downstream BIA caused by inflammation. Deferiprone has shown promise in treating PD, reducing iron content in the caudate and dentate nuclei (192). Deferiprone shows efficiency at reducing tau phosphorylation and Aβ accumulation in a rabbit model (193). An issue with iron chelators is that because they must be delivered systemically, they are likely to affect iron levels in the systemic circulation. This would be damaging to elderly populations and those with chronic disease due to the anemia and iron deficiency seen in aging and inflammatory disorders. Treating with an iron chelator would be tackling the secondary harmful event, the iron accumulation, rather than the inflammation itself. A primary limitation of iron chelators such as deferiprone and deferasirox (Exjade^@^) is their affinity for iron. The stability constants of their complexes with iron exceed 10^25^, a value that indicates either would sequester essentially all of the iron in a cell or organism. Thus, while useful in the treatment of iron overload as in hemochromatosis (194), they are contraindicated for the management of the progressive iron accumulation downstream of chronic inflammation (195). A cautionary example is in the use of deferiprone for treatment of Friedreich's Ataxia, an autosomal recessive disorder resulting in the reduction of frataxin essential to the function of all iron-containing enzymes in the mitochondria. In this disease, iron accumulates in the mitochondria, a pathology logically addressed by iron chelation. Not surprisingly, deferiprone treatment exacerbates the iron enzyme deficiency, out-competing the cell's iron metabolism machinery for this essential co-factor (196).

Clearly, iron chelation and anti-oxidant approaches have proven frustrating in that the results have been variable, and in some cases, contra-indicating. On the other hand, there is accumulating evidence that a combination therapy may hold promise. For example, combination of deferiprone and N-acetyl cysteine was efficacious in suppression of the brain iron accumulation, mitochondrial dysfunction and reduced dendritic spine density in mice with chronic brain iron overload (197). An excellent argument has been given as to why managing neuronal iron and redox biology is likely to be a productive pharmacologic approach (198). Indeed, recent reports of the efficacy of a quinazolinone derivative, PBT434, in a Phase I trial involving Multiple System Atrophy indicate the potential of such chemical platforms (199). PBT434 suppresses α-synuclein toxicity in models of Parkinson's disease (200) and MSA (201). PBT434 has a moderate affinity for both ferrous and ferric iron (stability constants ~10^7^); has a redox potential that supports cell anti-oxidant activity; and exhibits facile equilibration across the BBB without disrupting iron homeostasis in BMVEC (200, 202). In short, PBT434 reflects the chemical properties that appear most desirable in a rationally developed pharmaceutical approach to managing the cell dysfunction associated with BIA.

Ferroptosis is a more recently identified form of iron-dependent cell death. This type of cell death is induced by RAS-selective lethal compounds (RSLs), which were found to be selectively lethal to oncogenic RAS-mutant cell lines. Ferroptosis is divergent from other apoptotic pathways because it is associated with increased levels of ROS and is prevented by iron chelation, suggesting dependence on iron and oxidative stress. Erastin and RSL3, two important RSLs, can induce oxidative, iron dependent cell death (203). Glutathione can prevent this form of cell death, as depletion of a glutathione peroxidase and subsequently glutathione is a mechanism behind ferroptosis induction (204). Deferoxamine, another high-affinity iron chelator, is also efficient at preventing ferroptosis (205). As inflammation can result in oxidative stress, it is possible that inflammation-induced BIA can result in cell death by ferroptosis, but more research is needed to confirm this. If ferroptosis is induced by inflammation, glutathione and iron chelation may work as therapeutics for the resulting BIA.

Of course, all of the above pharmacologic strategies address the consequences of inflammation-dependent BIA and not the inflammatory process nor the mechanism(s) underlying the BIA itself. In regard to the latter, the fact is that the brain's iron content *does* increase. This can result from an increase in iron trafficking from the circulation at the BBB; a decrease in the rate of diffusive iron efflux at the cerebrospinal fluid barrier; or a combination of these changes in metabolite flux. This review has focused primarily on the latter reflecting the fact that accumulating evidence supports the premise that both transcellular and paracellular, apical to basolateral brain iron uptake *is* increased in an inflammatory state (Figure 3). This model suggests alternatives to the iron chelation and anti-oxidant approaches, namely, modulation of the activity of the iron efflux protein, FPN, and maintenance of the tight junctions that suppress paracellular blood-to-brain leakage.

A rational way of modulating the functional activity of Fpn is *via* managing its residence time in the plasma membrane. As noted, FPN membrane occupancy is regulated by its interaction with hepcidin. Systemic hepcidin regulates the function of FPN found at the apical, blood membrane of all cells, including BMVEC; glial cell-secreted hepcidin regulates FPN basolateral localization (Figure 3). Thus, conceptually, a hepcidin antagonist functions to up-regulate apical FPN-dependent BMVEC iron efflux while a basolateral hepcidin agonist would suppress iron efflux into the abluminal space. Both such compounds are in development. For example, a Phase I trial of an antagonist in the treatment of patients with an anemia caused by elevated circulating hepcidin has shown considerable clinical benefit (206). Such treatment increases the flux of iron *into* circulation from cells, *eg*, apical iron efflux *from* BMVEC. With respect to hepcidin “agonists” the most promising are not agonists, *per se*, but hepcidin-mimics, “mini-hepcidins” (MH) (207, 208); MH are poly-peptides including 7–10 residues of the hepcidin sequence. Several have been used in pre-clinical studies (209, 210) including a recent report of improvement in a mouse model of β-thalassemia major (211). The objective of targeting a hepcidin agonist to the basolateral-localized FPN—indeed, the FPN localized to any membrane facing the brain's interstitium—is its delivery across the BBB. The molecular bases for pursuing this rational pharmaceutic are in hand given the extensive study of both MH and methodologies for accessing the abluminal space.

Clearly, the mechanisms underlying inflammation-induced BIA and its link to neurodegenerative disorders remain a critical area of research in two areas: molecular cell and correlated animal model studies to interrogate the mechanisms underlying the increase in brain iron specifically; and use of these experimental paradigms to develop a mechanistic understanding underlying the efficacy of small molecules that support a remediation of the cell misfunction revealed in these model studies. One objective of this review was to provide likely targets for this future drug discovery. The bottom line is that, at present, there are no governmentally-approved pharmacologic approaches for the treatment of BIA or the associated neuronal pathology.

## Author Contributions

SR: conceptualization, methodology, validation, investigation, and writing-review and editing. SR and DK: writing-original draft preparation and supervision. DK: project administration, funding acquisition, chronic inflammation, iron trafficking, brain iron, neurodegeneration, aging, and blood-brain barrier. All authors contributed to the article and approved the submitted version.

## Funding

This work was supported by an award from the National Institutes of Health of the United States of America (Award NS102337) to DK.

## Conflict of Interest

The authors declare that the research was conducted in the absence of any commercial or financial relationships that could be construed as a potential conflict of interest.

## Publisher's Note

All claims expressed in this article are solely those of the authors and do not necessarily represent those of their affiliated organizations, or those of the publisher, the editors and the reviewers. Any product that may be evaluated in this article, or claim that may be made by its manufacturer, is not guaranteed or endorsed by the publisher.

## Abbreviations

BIA: Brain Iron Accumulation
AD: Alzheimer's Disease
PD: Parkinson's Disease
MSA: Multiple System Atrophy
MS: Multiple Sclerosis
APPs: Acute Phase Proteins
BBB: Blood Brain Barrier
BMVECs: Brain Microvascular Endothelial Cells
TBI: Transferrin Bound Iron
NTBI: non-transferrin bound iron
Tf: Transferrin
TfR: Transferrin Receptor
DMT1: Divalent Metal Transporter 1
FPN: Ferroportin
APR: Acute Phase Response
CRP: C-Reactive Protein
AI: Anemia of Inflammation
HO-1: Heme Oxygenase-1
ROS: Reactive Oxygen Species
SWI: Susceptibility Weighted Imaging
QSM-MRI: Quantitative Susceptibility Mapping MRI
EAE: Experimental Autoimmune Encephalomyelitis
6-OHDA: 6-hydroxydopamine
HIF-1α: Hypoxia Inducible Factor 1α
TJs: Tight Junctions
AJs: Adherens Junctions
ZO-1: Zona-occludens 1
HUVECs: Human Umbilical Vein Endothelial Cells
BRB: Blood Retinal Barrier
VEGF-A, Vascular Endothelial Growth Factor: A
CFA: Complete Freund's Adjuvant
DCE-MRI: Dynamic Contrast Enhanced MRI
NAC: N-acetyl cysteine
NSAIDS: non-steroidal anti-inflammatory drugs
APP: Amyloid Precursor Protein
RSLs: RAS-selective lethal compounds
MH: Mini Hepcidins.
